# Embolization for Type Ia Endoleak after EVAR for Abdominal Aortic Aneurysms: A Systematic Review of the Literature

**DOI:** 10.3390/biomedicines10061442

**Published:** 2022-06-18

**Authors:** Elena Marchiori, Abdulhakim Ibrahim, Johannes Frederik Schäfers, Alexander Oberhuber

**Affiliations:** Department of Vascular and Endovascular Surgery, University Hospital Münster, 48149 Münster, Germany; abdulhakim.ibrahim@ukmuenster.de (A.I.); johannes.schaefers@ukmuenster.de (J.F.S.); alexander.oberhuber@ukmuenster.de (A.O.)

**Keywords:** aneurysm, endovascular aneurysm repair, embolization, endoleak, Ia, proximal, liquid embolic agent, coils, safety, outcome

## Abstract

(1) Successful endovascular repair for abdominal aortic aneurysms is based on the complete exclusion of the aneurysm sac from the systemic circulation. Type Ia endoleak (ELIA) is defined as the persistent perfusion of the aneurysm sac due to incomplete proximal sealing between aorta and endograft, with a consequent risk of rupture and death. Endoleak embolization has been sporadically reported as a viable treatment for ELIA. (2) A systematic literature search in PubMed of all publications in English about ELIA embolization was performed until February 2022. Research methods and reporting were performed according to the Preferred Reporting Items for Systematic Reviews and Meta-Analyses (PRISMA) statement. Data regarding patient numbers, technical success (endoleak absence at control angiography), reinterventions, clinical and imaging follow-up, and outcomes were collected and examined by two independent authors. (3) Twenty-one papers (12 original articles, 9 case reports) reported on 126 patients (age range 58–96 years) undergoing ELIA embolization 0–139 months after the index procedure. Indication for embolization was most often founded on unfavorable anatomy and patient comorbidities. Embolic agents used include liquid embolic agents, coils, plugs and combinations thereof. Technical success in this highly selected cohort ranged from 67–100%; the postprocedural complication rate within 30 days was 0–24%. ELIA recurrence was reported as 0–42.8%, with a secondary ELIA-embolization-intervention success rate of 50–100%. At a follow-up at 0–68 months, freedom from sac enlargement amounted to 76–100%, freedom from ELIA to 66.7–100%. (4) Specific literature about ELIA embolization is scant. ELIA embolization is a valuable bailout strategy for no-option patients; the immediate technical success rate is high and midterm and long-term outcomes are acceptable.

## 1. Introduction

Abdominal aortic aneurysm (AAA) is a pathological enlargement of the aorta, which can cause rupture and profuse internal bleeding that can be lethal without urgent treatment. Indication for aneurysm repair is given by progressive sac enlargement (>5 mm within six months), eccentric aneurysms and aneurysm sac size > 50–55 mm, with tendentially a lower indication cut-off for women (50 mm) [1]. Options for treatment consist of open surgical repair (OR) or endovascular aneurysm repair (EVAR), the kind of management being chosen depending on anatomy, comorbidity, life expectancy and estimated perioperative mortality of the patient, as well as on patient preference. With EVAR, a Y-shaped-endograft is percutaneously implanted in the aorta with its endings landing in a healthy zone of the vessels proximal and distal of the aneurysmatic dilatation. Subsequently, blood flow is channeled within the stent-graft, discharging the pathological vessel segment from pressure.

**Figure 1:**
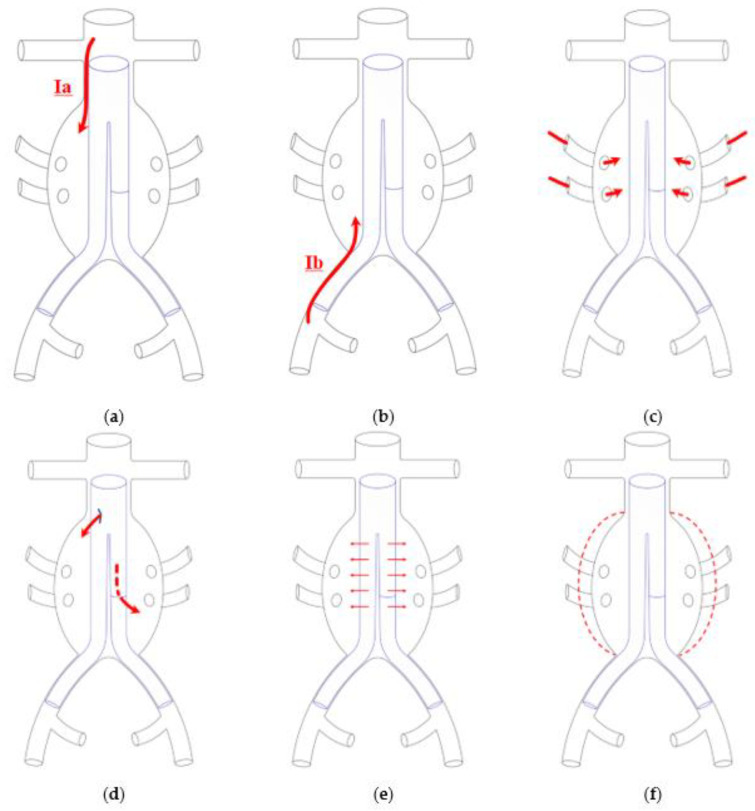
Schematic representation of endoleak types. (**a**) Type Ia endoleak (ELIA), originating from the proximal end of the stent-graft; (**b**) Type Ib endoleak, originating from the distal end of the stent-graft; (**c**) Type II endoleak, originating from retrograde flow from the inferior mesenteric artery or lumbal arteries; (**d**) Type III endoleak, due to stent-graft component disconnection or fabric tear; (**e**) Type IV endoleak, due to stent-graft material porosity; (**f**) Type V endoleak, due to unidentified source, sac expansion without visible any visible leak.

The long-term success of EVAR is based on the complete exclusion of the aneurysm sac from systemic circulation. Endoleaks consist of flow presence in the aneurysm sac outside the graft and are one of the most crucial complications after EVAR [2,3]. They are classified into five different types according to the source of the leak (Figure 1).

EVAR is increasingly used, particularly for elderly and comorbid patients [4,5], with a trend to device implantation at the limit or outside the instructions for use (IFU), with consequent increased risk of endoleak and related failure of aneurysm exclusion [6]. The incidence of type I endoleak after EVAR is estimated at 5% during 5-year follow ups [3].

**Figure 2:**
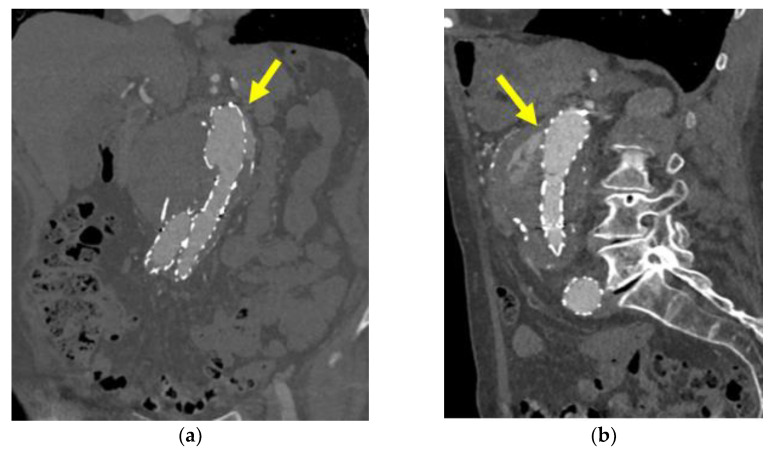
CTA demonstrating a type Ia endoleak after infrarenal endovascular aneurysm repair for AAA: (**a**) coronal and (**b**) sagittal views demonstrate the contrast flow (yellow arrow) within the aneurysm sac entering from the proximal end of the stent-graft; CTA, computed tomography angiography; AAA, abdominal aortic aneurysm.

Type Ia endoleak (ELIA) is defined as the persistent perfusion of the aneurysm sac due to incomplete sealing at the proximal aortic attachment site [2] (Figure 1a); this clinical entity is associated with sac pressurization and subsequent risk of rupture and death and should therefore be treated promptly [2,3]. The gold standard for ELIA detection currently is computed tomography angiography (CTA), which allows detailed leak visualization and treatment planning (Figure 2). Proximal sealing is assured by the radial force of metallic stent frames in most of the available stent-grafts, or by polymer filled O-rings in some endografts (i.e., Ovation Endograft, Endologix, Irvine, CA, USA) [7].

Multiple strategies have been suggested for achieving proximal sealing, and different therapeutic options are available according to anatomical features. Conventional treatment strategies for ELIA include balloon dilatation, bare metal stents, endostaples and proximal cuffs; rising in complexity, endovascular procedures extending to the proximal segment of the aorta which implicate the cannulation of the visceral vessels in the stent-graft configuration (fenestrated-EVAR, FEVAR; chimney-EVAR, ch-EVAR; branched-EVAR, BEVAR) can be considered. Conversion to open surgery can be performed with acceptable results in selected fit patients [8]. Endoleak embolization has been reported as a viable treatment for ELIA, in particular for patients where none of the aforementioned options are available, however, most of the existing data arise from small cohorts. The present systematic review aims to recapitulate the existing data for embolization in the treatment of ELIA and summarizes the evidence concerning safety, efficacy, outcome and follow-up.

## 2. Materials and Methods

### 2.1. Data Sources and Search Strategy

A systematic literature review was performed in accordance with the Preferred Reporting Items for Systematic Reviews and Meta-Analyses (PRISMA) statement [9]. The scientific publications about embolization of ELIA for abdominal aortic aneurysms were searched for in the PubMed, Web of Science and Scopus databases. Reference lists of the articles were also examined to add relevant studies. The last search was conducted on February 2022. The query was performed with the strings “endoleak AND (type I[ti] or type 1[ti] or type Ia[ti] or type 1a[ti])”. The database query was conducted independently by two authors (E.M. and A.O.), and controversies were resolved by collegial discussion.

### 2.2. Study Eligibility Criteria, Study Quality Assessment

All articles and case reports dealing with treatment for ELIA or with the outcome of interventions for endoleaks after EVAR for abdominal aortic aneurysms were included. Inclusion criteria were (1) reporting in English, (2) reporting on EVAR for AAA, (3) reporting on ELIA or proximal endoleak with development during follow-up or persistence of an intraoperative ELIA after EVAR and (4) reporting on outcome data about the embolization (at least completion-angiography). Exclusion criteria were (1) reporting on ELIA in other aortic segments, in particular thoracic endovascular aneurysm repair (TEVAR) or fenestrated endovascular aneurysm repair (FEVAR) (e.g., aortic arch, thoracic aorta, thoracoabdominal aorta); (2) reporting on non-EVAR interventions, e.g., Endovascular Aneurysm Sealing (EVAS) and Multilayer Aneurysm Repair System (MARS); (3) articles with unspecified type of endoleak or not mentioning the number of ELIA; (4) articles reporting on “thrombization technique”, with fibrin glue filling of the aneurysm sac during index EVAR; (5) experimental studies, studies in vitro, commentary, letters to the editor; (6) full text not in English. Due to the paucity of data on this topic, articles describing spurious cohorts with both suitable and unsuitable patients (e.g., endoleak Ia and Ib, EVAR and EVAS, EVAR and FEVAR/TEVAR) were included as long as patients meeting the exclusion criteria did represent a minority in the cohort and the number of patients with ELIA was specified. The methodological quality of the studies was assessed using the Johanna Briggs case tool, critical appraisal checklist for case series, in consideration of the small number of patients described in each paper [10].

### 2.3. Data Extraction and Statistical Analysis

Data regarding study characteristics, patient numbers and demographics, treatment indication and timing of the embolization, aneurysm morphology, endoleak characteristics, intervention, outcome and follow-up were independently extracted and assessed by two authors (E.M. and either J.F.S. or A.I.). Disagreements were resolved by plenary discussion and a consensus was reached. Studies were considered retrospective if not otherwise specified. In spurious cohorts, if the data of single ELIA patients were available, only these were specifically collected and reported. All undescribed data where classified as “not available”. Study and patient characteristics, as well as procedure details, safety and follow-up data were collected and analyzed with Microsoft Excel (Microsoft Corporation, Redmond, WA, USA). Data are presented as ranges. Because individual patient data were not described in most papers, no meta-analysis was performed. None of the studies was randomized and none reported complete raw data, while some articles reported a selection of single-patient data; as a result, a meta-analysis could not be planned.

### 2.4. Endpoint Definition

For assessment of the safety and efficacy of embolization of ELIA, specific parameters were collected. The first endpoint was to assess the safety of embolization in ELIA. For this aim, data regarding complications < 30 days postoperatively, procedure-related complications, embolization-related complications and mortality within 30 days were extracted. The second endpoint was to summarize the efficacy and outcome of embolization at short-, mid- and long-term follow up. For this purpose, we defined the following parameters before data extraction: technical success, defined as the absence of ELIA at the completion angiography; freedom from endoleak, defined as absence of ELIA in follow-up imaging; freedom from sac enlargement, defined as sac shrinkage or stable sac size (growth < 5 mm). All parameters were collected in a table with the relevant institutional protocol. Follow-up methods and protocols, such as computed tomography (CT), CTA, aortic ultrasound imaging (US), contrast-enhanced ultrasound (CEUS) and magnetic resonance angiography (MRA), are reported in the tables. Data regarding reinterventions, long-term outcomes including survival, aneurysm-related death and ruptures, were collected whenever available.

## 3. Results

### 3.1. Search Results

**Figure 3:**
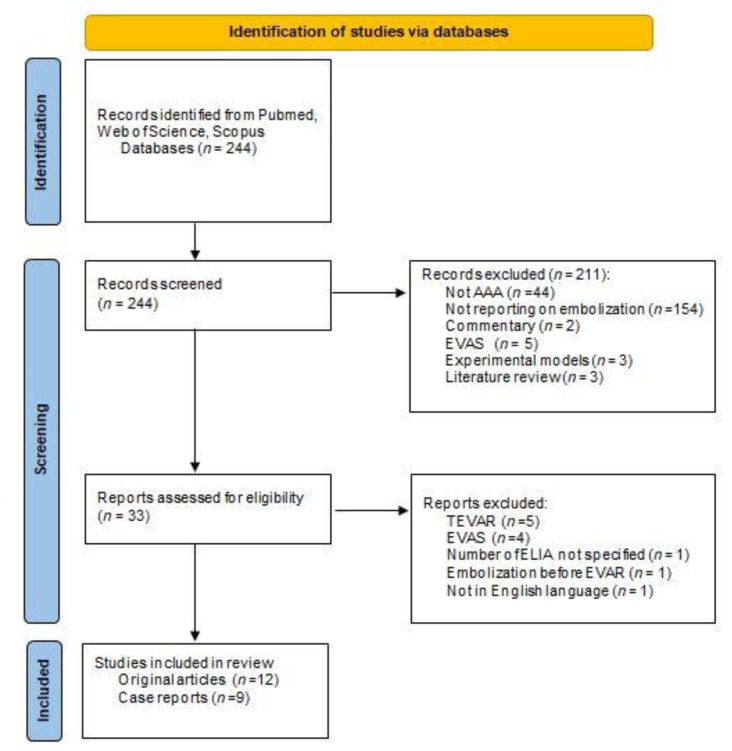
Flow diagram of the systematic search, study screening and inclusion according to the Preferred Reporting Items for Systematic Reviews and Meta-Analyses (PRISMA) statement [9].

Our initial search strategy retrieved 244 records, after removal of the duplicates and screening of title and abstract, 33 full-text articles were assessed for eligibility. The review was registered in PROSPERO (ID CRD42022333322). The flow diagram of the systematic search and selection is presented in Figure 3. Finally, 21 papers (12 original articles, 9 case reports) were included; examination of the references did not produce additional records [11,12,13,14,15,16,17,18,19,20,21,22,23,24,25,26,27,28,29,30,31]. None of the studies was randomized and none reported complete raw data, while some articles reported a selection of single-patient data; as a result, a meta-analysis could not be performed.

### 3.2. Patients and Index-Procedure Characteristics

Overall, 21 studies, corresponding to 12 original articles and 9 case reports, were analyzed: two papers reported data from a prospective longitudinal database [12,17], nineteen articles reported on retrospective data. These studies described 147 patients treated for type I endoleak, of these, 126 were type Ia endoleaks.

For original articles, patient demographics, cohort characteristics and indication to treatment are summarized in Table 1, for case reports, in Table 2. In the original articles, age range was 58–96 years, most of the patients were male (range 57.1–100%); three papers reported exclusively on ELIA [20,21,22], ten on different kinds of endoleaks. Six cohorts reported data beyond EVAR, i.e., TEVAR or FEVAR (*n* = 5 papers) [15,16,17,18,20], EVAS (*n* = 2 paper) [18,22] or MARS (1 paper) [17].

Endoleak embolization was performed in an elective setting in 57% (*n* = 12/21) of the papers, in urgent treatment in 10% (*n* = 2/21) and in both in 19% (4/21); 14% (*n* = 3/21) were unspecified. 

Sixteen authors (76%) report performing embolization exclusively during reinterventions, four (19%) both during index-procedure and reinterventions, one case report (1/21, 5%) and none of the case series reported on routine use of embolization solely during index procedure. Indication for embolization was most often based on unfavorable anatomy for other treatments (71%, 15/21 articles) and patients’ comorbidities (62%, 13/21). In addition to these, most of the authors stated the indication on the basis of multiple characteristics including endoleak persistence (33%, 7/21) and sac enlargement (6/21, 29%). The indications were based on a multidisciplinary decision in 14% of the reports (3/21) and on aneurysm rupture or urgent treatment in 14% (3/21). In single cases, the indication was based on patient’s preference, surgeon preference, routinely performed ELIA persisting during index procedure, or due to the need of anticoagulant treatment. For the quota of patients with a history of EVAS, one author reported embolization as the primary treatment for endoleaks type I [18].

Maximum aneurysm diameters ranged from 53 to 129 mm. For original articles, patient demographics, cohort characteristics and indication for treatment are summarized in Table 1, for case reports in Table 2.

### 3.3. Embolization of ELIA

The time interval between index procedure and embolization ranged from 0–139 months. In four studies and in a case report, a quota of the patients (17–53%) underwent embolization during the index procedure [13,15,16,22,31].

Five different ELIA approaches were described, the most used was transarterial embolization accessing the femoral artery (13/21, 62%) or the brachial/radial artery (6/21, 29%), two studies report on a percutaneous transabdominal approach [14,19], one on a translumbar approach [21] and two case reports on a transcaval approach [28,30].

Embolic agents used include liquid embolic agents (LEA), coils, plugs and combination thereof. LEA used involved n-butyl-cyanoacrylate (NBCA) and ethylene vinyl alcohol copolymer (EVOH) dissolved in dimethyl sulfoxide, commercially known as Onyx©.

The use of coils and LEA was reported in 81% (17/21) and 66% (14/21) of the papers, respectively, alone or in combination with other embolic materials. Two papers report plug use, mostly used in aiming to close specific outflow vessels. Eleven authors (11/21, 52%) report on adjunctive procedures (cuff, proximal extension, cuffs, chimney, endoanchors) performed as a previous intervention and/or prior to embolization, however, data regarding this aspect are reported discontinuously.

Technical success, defined as absence of ELIA in the control angiography at the end of the procedure, ranged from 67 to 100%. For original articles, procedure characteristics, materials and technical success are reported in Table 3, for case reports in Table 4.

Overall complications within 30 days ranged from 0–24%, major irreversible complication from 0–16.6%, minor complications from 0–14.3% and procedure related complications from 0–12%. The most common procedure-related complications are reported to be stent-grafts occlusions and LEA dislocation. In three cohorts, a single case of in-hospital mortality within 30 days was reported (due to colon ischemia with sepsis, multiorgan failure and acute coronary syndrome, respectively), generating an in-hospital mortality rate of up to 14%.

ELIA recurrence was reported in six series with a frequency amounting to 42.8%, and secondary embolization interventions were reported in five cohorts, with a success rate in reinterventions ranging from 50–100%. Two cohorts described patients which underwent open conversion because of sealing failure [13,22], and three described patients with recurrence but without reintervention or other treatment in the sense of expectant or palliative management [13,18,22]. Data regarding post-procedural events, recurrences and reinterventions after ELIA embolization are shown in Table 5.

### 3.4. Endpoints, Outcomes and Follow-Up

Excluding missing data, endpoints for evaluating ELIA-embolization outcomes in a case series were based on the absence of endoleak in the completion angiography, measurements of aneurysmal sac maximal diameter, and absence of endoleak on imaging, or a combination of these. Follow-up methods comprised CTA and duplex, and in some studies non-contrast CT or MRT limited to patients with contraindications for the other methods. One case series and one case study report contrast-enhanced ultrasound (CEUS) [21,27]. Follow-up length was reported in all studies and ranged from a few days to 68 months. Most of the case series report a follow-up protocol based on CTA within one or three months, at 6 months, at 12 months, and annually thereafter. Freedom from sac enlargement amounted to 76–100%, freedom from ELIA ranged from 66.7 to 100%. Three cases of conversion to open surgery after ELIA embolization (3/126, 2.4%) were reported.

Data regarding secondary aneurysm rupture are reported in five papers. Overall in the whole literature overview, a total of 7 patients (7/126, 5.6%) presented aneurysm rupture at follow-up, with the timing varying from 2.5 to 18 months after the intervention. Survival data are not systematically reported. Seven authors describe a total of 16 not-aneurysm-related deaths at follow-up, and one author presented data survival rates amounting to 72%, 65% and 49% at 1, 2 and 4 years, respectively [22]. Data regarding outcomes and follow-up events are shown in Table 6.

## 4. Discussion

The present systematic review included 21 publications (12 case series, 9 case reports) reporting on 126 patients with ELIA. Indication for embolization was most often based on unfavorable anatomy and patient comorbidities, immediate technical success ranged from 67 to 100%. The postprocedural complication rate within 30 days was 0–24%. Embolization offers the advantage of a minimal invasive procedure and is a valuable tool for a highly selected patients’ collective which can’t undergo open conversion and/or more complex endovascular procedures involving the visceral vessels.

At follow-up, freedom from sac enlargement amounted to 76 to 100%, freedom from ELIA to 66.7 to 100%. This suggests that ELIA embolization can help to achieve sealing and avoid rupture in the midterm for patients with a lack of alternatives, while it remains necessary to perform strict follow-up. The residual risk of ELIA recurrence and aneurysm rupture remains one of the significant limitations of this bail-out option. Current data regarding the efficacy and midterm outcome of ELIA embolization are limited and restricted to miscellaneous case series, including 3 to 23 patient and case reports. No prospective register was found. Two papers retrospectively analyzed the data of prospectively conducted EVAR databases. Overall, most cohorts were heterogeneous; only three papers reported specifically on ELIA [20,21,22]. In six articles, the type of index-procedure was spurious, with data derived from non-EVAR patients (i.e., TEVAR, FEVAR, EVAS, MARS) [15,16,17,18,20,22], precluding the possibility of a meta-analysis.

EVAR is a common treatment method for infrarenal AAA and is increasingly used particularly for elderly and comorbid patients [4,5]. Moreover, the trend for using implantation at the limit or outside IFU has been proven to lead to an increased risk of ELIA and a related failure of aneurysm exclusion [6].

None of the papers systematically reported the etiology of ELIA, and no data on the quota of non-IFU implantation were available, although cases of hostile anatomy, stent-graft migration and neck degeneration were occasionally reported.

In the reviewed literature, the time interval between index procedure and embolization ranged from 0–139 months, showing that ELIA embolization was performed in immediate as well as in delayed leaks. Regarding immediate leaks, in five papers 17–53% of the patients underwent embolization after ELIA detection at completion angiography during the EVAR procedure [13,15,16,22,31].

ELIA manifesting during the EVAR procedure has been described to occur in relation to adverse anatomy, morphology outside the company´s instruction of use (IFU) and malpositioning or undersizing of the stent-graft. Studies have proven that short angulated proximal neck zones in particular can lead to ELIA [32]. In delayed ELIA progression of aneurysmal disease, degenerative neck dilatation and migration of the stent-grafts are alleged to play the main role. Implantation by aneurysm morphology outside IFU parameters has been proven to also have an incremental negative effect on long term results [33], and to increase the frequency of sac expansion during follow-up [6].

Twelve authors (12/21, 57%) reported performing embolization exclusively in an elective setting, and sixteen authors (76%) reported performing during a planned reintervention, highlighting the existence of a selected patient cohort for which conventional therapy methods are nearly exhausted. This is coherent with the finding that the indication for embolization was most often based on a proximal neck anatomy unsuitable for other procedures (71% of the authors) and patient comorbidities (62% of the authors). Other frequent indications were ELIA persistence (33% of the authors), sac enlargement (29%), multidisciplinary decision (14%) or urgent treatment (14%).

More than the half of the authors (11/21, 52%) reported on adjunctive procedures (infrarenal extension cuffs, device relining, chimney, endoanchors) performed as a previous intervention or before embolization, in 14.3–54.5% of the patients. Some authors explicitly stated to have performed these procedures whenever possible. The analysis of data regarding the indication conjunct with the data regarding adjunctive procedures highlights that most ELIA were primarily treated as far as achievable with regular therapies (in particular with balloon molding, proximal extensions and endoanchors), and suggests that ELIA embolization was used as bail-out option for complex cases.

Embolization was most often performed through a minimally invasive procedure with a percutaneous transarterial approach through the femoral (62% of the authors) or brachial/radial artery (29% of the authors). Access to the aneurysm sac and ELIA was, however, also gained with transabdominal, translumbar and transcaval approaches [21,28,30], these options being particularly advantageous when the arterial pathway failed.

Between embolic agents, the use of coils and LEA wase predominant, and were reported in 81% (17/21) and 66% (14/21) of the papers, alone or in combination with other embolic materials. Two papers reported on plug use, these proving effective in closing specific outflow pathways.

All but one case series described a technical success rate greater than 80%, and most often amounting to 100%. This is reasonable, considering that the treatment of ELIA is deemed mandatory, and in the clinical practice, every effort is made to seal ELIA before finishing the interventions. Marcelin et al. [20] reported specifically on gutter endoleak, and, interestingly, obtained 67% immediate technical success but a comparable quota of freedom-from-sac-enlargement and endoleak recurrence at follow-up compared to other authors. This observation sustains the hypothesis that gutter endoleaks could undergo different etiologies and have a different prognosis in the progression of aneurysmal disease in comparison with other ELIA. 

Regarding safety, overall complication rate < 30 days was 0–24%, and procedure-related complications were reported at 12%. The most common procedure-related complications were stent-grafts occlusions and LEA dislocation, evidencing that ELIA embolization is technically challenging, and in particular that the controlled release of both LEA and coils requires expertise. Although ELIA embolization is a minimally invasive procedure, minor (reversible) and major (irreversible) complications have been reported. These consist most often of puncture site hematomas, acute renal failure and stroke or acute coronary syndrome, reflecting the cardiovascular and comorbid high-risk profile of the patients. Major irreversible complication amounted to 0–16.6%, and stent-graft occlusion and LEA dislocation occurred in up to 9% of the patients. In three cohorts, a single case of in-hospital mortality within 30 days was reported, due to colon ischemia with sepsis, multiorgan failure and acute coronary syndrome, respectively. Because of the small cohort size, single events generated an in-hospital mortality rate of up to 14%.

Follow-up plays a pivotal role for the efficacy and safety of ELIA treatments, because this entity presents symptoms only in the life-threatening development of an aneurysm rupture. ELIA recurrence was reported in six series, with a frequency amounting to 42.8%. However, with prompt secondary embolization interventions, complete ELIA sealing was achieved for 50–100% of the patients. 

Two cohorts described patients which underwent open conversion because of sealing failures [13,19], and three described patients with recurrence but without reintervention or other treatment in the sense of expectant or palliative management [13,18,22]. 

Regarding recurrences, this literature overview highlights two important prognostic aspects of ELIA natural history: first, some patients originally deemed unsuitable for open conversion or complex endovascular procedure underwent these, evidencing that the risk–benefit balance for persistent endoleaks can lead to the utilization of riskier strategies with success; secondly, some patients can end up in a palliative situation, in acknowledgement that that no further therapeutic option is available.

Follow-up and lifelong imaging surveillance are mandatory according to international guidelines [2,3]. Described endpoints after ELIA embolization refer to the absence of endoleaks in the completion angiography, serial measurements of aneurysmal sac maximal diameter and absence of endoleaks on imaging, or a combination of these. Follow-up length was reported in all studies and ranged from a few days to several years (0–68 months). Most institutional protocols were based on CTA within one or three months, at 6 months, at 12 months and annually thereafter. Follow-up imaging is crucial for assessment of the efficacy of ELIA seal. CTA is considered the gold-standard for ELIA detection, and because of reproducible aneurysm-sac-diameter measurements, it is, however, associated with an increased burden of radiation and acute kidney injury [34,35]. MR, non-contrast CT and CEUS were sporadically reported. The maximum diameter of the aneurysm, measured perpendicular to the center-line reconstruction or on multiplanar reformation, remains the main parameter for predicting the risk of sac rupture, and can also be assessed in non-contrast CT, MR and, with limited reproducibility, in duplex. After embolization with LEA, duplex and CEUS offer the advantage of biasing the problem of embolization-correlated artifacts, and can effectively identify ELIAs. However, regarding sac size, reproducibility is limited. Overall, the data from the current systematic review reported freedom-from-sac-enlargement amounting to 76–100%; freedom from ELIA ranged from 66.7 to 100%.

A loss of sealing after ELIA embolization can lead to aneurysm rupture. Therefore, prompt diagnosis and treatment are mandatory. ELIA recurrence is initiated by a failure of the circumferential seal between stent, embolic agents and the aneurysm. The sac is therefore further directly perfused through the circulation-acquiring systemic pressure and generates high tension on the aortic wall [36]. Overall in the described literature, 5.6% of the ELIA evolved into rupture after embolization treatment (7/126, 5.6%); five of these seven patients presented with rupture within 6 months, and two after more than one year. In some cases, the endoleak recurrence was known but not treated due to patients’ refusal of further therapies. Overall, in the described literature three patients (2.4%) underwent conversion to open surgery.

Survival data were not systematically reported. The available data suggest that this patient cohort is burdened by severe comorbidities implying high rates of not-aneurysm-related deaths at follow-up. One author presented data with estimated survival rates in the Kaplan–Meier curve at 4 years amounting to 49%.

ELIA embolization is technically challenging. The application of LEA and coils requires expertise, and an accurate identification of the endoleak source is essential for achieving a complete seal. All patients treated with ELIA embolization should be routinely monitored in order to promptly identify signs of recurrence and sac expansion, and consequently, therapeutic strategy escalation should be considered before fatal complications occur. Treatment indications (endoleak persistence, sac enlargement, multidisciplinary decision, aneurysm rupture or urgent treatment) highlight a selected patient cohort with mostly no option for other treatment, on the basis of one or more of these issues. The existence of a subgroup of patients with a lack of treatment alternatives is confirmed by the reports of palliative follow-up because of “no-options” situations, in which all therapeutic options were exhausted regarding the patient’s characteristics. This aspect emerges not only due to the anatomy, but also, and in particular, in the context of comorbidities.

Overall, embolization is a minimally invasive procedure, available off-the shelf also in emergency settings, which allows experienced operators to treat complex ELIA with lower contrast-medium and radiation exposure in comparison with complex endovascular repair. It can be performed through a transarterial access and treat a wide variety of aortic morphologies, representing a valuable option for patients lacking any other therapeutic options. The preservation of the proximal native aortic segment allows escalating the therapy with complex aortic stent-grafts in case of recurrence, or to gain time before a second step for patients needing a recovery phase, or in urgent settings, when awaiting the production of complex custom-made stent-grafts.

Generating strong recommendations with the available data is complicated by the heterogeneous cohort composition as well as by the variety in timing and indications of the reported embolization procedures. The collection of prospective, exhaustive data with means of multicentric registry for further validation of the results is required. 

## 5. Conclusions

Embolization for the treatment of type IA endoleaks after EVAR is a valuable bail-out strategy for no-option patients with hostile neck anatomy and severe comorbidity, contraindicating complex endovascular repair and conversion to open surgery. It presents a high primary immediate success rate, a low rate of periprocedural complications and acceptable midterm outcomes. Due to the risk of aneurysm, disease progression and ELIA recurrence, a strict follow-up protocol is mandatory.

## Figures and Tables

**Table 1:** Baseline data on included case series: type of study, patient demographics and indication for embolization.

Author	Year	Type of Study	N Type I	N Type Ia	Age Range (Mean) [Years]	Male Sex [%]	Elective or Urgent	TEVAR	FEVAR	ch-EVAR	EVAS	MARS	Indication	Index Procedure or Reintervention	Diameter (Mean) [mm]
Golzarian [11]	1997	R	7	3	58–80 *	100% *	E	0	0	0	0	0	EL SAC	Re	NA
Faries [12]	2003	P	8	7	76.4	86%	NA	0	0	0	0	0	UNSU UNFIT	Re	59–82 (64)
Maldonado [13]	2003	R	24	17	NA	83%	E	0	0	0	0	0	UNSU SAC EL SURG	both (53% index *)	NA
Choi [14]	2011	R	7	6	58–81 (69.3)	85.7%	both	0	0	0	0	0	UNSU UNFIT	Re	58–117
Henrikson [15]	2011	R	6	5	62–88 (77)	100%	NA	1	0	4	0	0	UNSU UNFIT	both (33% index)	70–93 (83)
Chun [16]	2013	R	6	4	62–82	83.3%	E	2	1	0	0	0	UNSU UNFIT	both (17% index)	NA
Eberhardt [17]	2014	P	8	7	68–86 *	57.1%	E	5	0	0	0	1	UNSU UNFIT	Re	NA
Ameli-Renani [18]	2017	R	25	23	64–96 (80)	80%	both	1	0	0	11	0	MULTI UNSU primary treatment for EVAS	Re	53–129 (82)
Graif [19]	2017	R	8	6	77–89 (78)	75%	E	0	0	0	0	0	MULTI UNSU UNFIT	Re	NA
Marcelin [20]	2017	R	9	9	62–87 (78.6)	66.7%	NA	1	0	9	0	0	UNSU UNFIT MULTI EL + SAC	Re	58–135 (81)
Ierardi [21]	2018	R	8	8	65–83 (72.5)	75%	both	0	0	NA	0	0	SAC RUP	Re	54–70
Marchiori [22]	2019	R	22	22	68–90 (77)	73%	both (23% urgent)	0	0	9	1	0	UNSU UNFIT SAC	both (18% index)	56–117 (74)

Legend: N, number of patients; EL, endoleak; NA, not available; R, retrospective study; P, prospective study/database; *, data specifically referring to ELIA patients; E, elective; TEVAR, thoracic endovascular aneurysm repair; FEVAR, fenestrated endovascular aneurysm repair; ch-EVAR, chimney endovascular aneurysm repair; EVAS, endovascular aneurysm sealing; MARS, multilayer aneurysm repair system; EL, endoleak persistence; SAC, aneurysm sac expansion; UNSU, anatomy unsuitable for other procedures; UNFIT, patient comorbidities; SURG, surgeon preference; MULTI, multidisciplinary case discussion and decision; RUP, aneurysm rupture; Re, Reintervention.

**Table 2:** Baseline data on included case reports: patient demographics and indication for embolization.

Author	Year	N Type I	N Type Ia	Age [Years]	Sex	Elective or Urgent	TEVAR	FEVAR	ch-EVAR	EVAS	MARS	Indication	Index Procedure or Reintervention	Diameter [mm]
Kirby [23]	2003	1	1	76	M	E	0	0	0	0	0	UNSU UNFIT	Reintervention	90
Peynicioglu [24]	2008	1	1	70	M	E	0	0	0	0	0	UNFIT EL	Reintervention	>110
Grisafi [25]	2010	1	1	92	F	U	0	0	0	0	0	UNSU SYMPT PAT	Reintervention	60
Loffroy [26]	2010	1	1	80	M	E	0	0	0	0	0	EL	Reintervention	NA
Arici [27]	2014	1	1	82	M	E	0	0	0	0	0	UNSU UNFIT	Reintervention	73
Gandini [28]	2015	1	1	82	M	U	0	0	0	0	0	UNFIT RUPT	Reintervention	57
Igari [29]	2016	1	1	77	M	E	0	0	1	0	0	UNSU SAC+EL	Reintervention	57
Massimi [30]	2017	1	1	77	M	E	0	0	1	0	0	EL	Reintervention	90
Belczack [31]	2019	1	1	72	NA	U	0	0	0	0	0	UNSU	Index	64

Legend: N, number of patients; NA, not available; M, male; F, female; E, elective; U, urgent; TEVAR, thoracic endovascular aneurysm repair; FEVAR, fenestrated endovascular aneurysm repair; ch-EVAR, chimney endovascular aneurysm repair; EVAS, endovascular aneurysm sealing; MARS, multilayer aneurysm repair system; EL, endoleak persistence; SAC, aneurysm sac expansion; UNSU, anatomy unsuitable for other procedures; UNFIT, patient comorbidities; PAT, patient preference; MULTI, multidisciplinary case discussion and decision; SYMPT, symptomatic aneurysm.

**Table 3:** Case series: embolization procedure characteristics, materials, approach and technical success.

Author	Time Interval Index to Embolization Procedure (Mean) [Months]	Approach	Embolic Agents	Adj. Type	Adj. %	Adj. Comments	Technical Success
Golzarian [11]	3–8 *	F B	Coils (1 ELIA + gelatin sponge)	0	0	-	100% *
Faries [12]	14.5 ± 5.7	A	Coils	0	0	-	100%
Maldonado [13]	NA	F	LEA (N) Coils	extender cuff	29.4%	Performed whenever possible	92.3%
Choi [14]	0–42 (9.6)	F T	LEA (N) Coils	extender cuff palmaz	33.3%	-	85.7%
Henrikson [15]	NA	F B	LEA (O) Coils	proximal extension	40%	-	100%
Chun [16]	0–72	A	LEA (O)	0	0	-	100%
Eberhardt [17]	0–108	F B	LEA (O) Coils	endoanchors	14.3%	-	100%
Ameli-Renani [18]	0–139 (22.5)	F B	LEA (O) Coils	0	0	-	100%
Graif [19]	1.6–106	F T	LEA (O) Coils Plugs	0	0	-	83.3% *
Marcelin [20]	3–15 (6.8)	F	LEA (O) Coils	chimney extensions	NA	chimney extensions	67%
Ierardi [21]	NA	F L	LEA (N,O) Coils	cuff	50%	-	100%
Marchiori [22]	0–84 (26)	B	O,C,P	cuff, endoanchors chimney extensions	54.5%	performed whenever possible	100%

Legend: A, arterial; F, percutaneous transarterial femoral; B percutaneous transarterial brachial/radial; T, transabdominal;L, translumbar; C, transcaval; LEA, liquid embolic agent; O, liquid embolic agent ethylene vinyl alcohol copolymer Onyx©; N, liquid embolic agent n-butyl-cyanoacrylate; Adj., adjunctive procedures; *, data specifically referring to ELIA patients.

**Table 4:** Case reports: embolization procedure characteristics, technical success and outcomes.

Author	Time Interval (Index to Embolization Procedure)	Approach	Embolic Agents	Adj. Type	Technical Success	Complications	Outcomes	Follow-Up Method	Months	Freedom from Sac Enlargement	Freedom from Endoleak	Recurrence rrence	Reinterventions	Ruptures
Kirby [23]	2 days	F	LEA (N)	palmaz extender cuff	yes	0	Angio EL	CTA	3	NA	NA	0	0	0
Peynicioglu [24]	11 days	F	LEA (N) Coils	0	yes	0	Angio, EL Sac	CTA clinical	12	NA	NA	0	0	0
Grisafi [25]	2 years	F	LEA (O)	palmaz extender cuff	yes	0	Angio	CT	12	NA	NA	0	0	0
Loffroy [26]	2 months	A	Coils	stent-graft extension	yes	0	NA	CTA	6	NA	yes	0	0	0
Arici [27]	3 months	F	Coils	0	yes	0	EL Sac	CTA CEUS	6	Yes	Endoleak II	0	0	0
Gandini [28]	7 months	C	Coils + thrombin	extender cuff	yes	temporary dialysis (recovery)	Angio EL Sac	NA	12	Yes	yes	0	0	0
Igari [29]	2 years	B	Coils	0	yes	0	EL Sac	duplex	3	Yes	NA	0	0	0
Massimi [30]	1 months	C	Coils	0	yes	0	Angio EL Sac	CTA	1	Yes	NA	0	0	0
Belczack [31]	intraoperative	NA	Coils	0	yes	0	Angio	clinical	0,1	NA	NA	0	0	0

Legend: A, transarterial; F, percutaneous transarterial femoral; B percutaneous transarterial brachial/radial; C, transcaval; LEA, liquid embolic agent; O, liquid embolic agent ethylene vinyl alcohol copolymer Onyx©; N, liquid embolic agent n-butyl-cyanoacrylate; Adj., adjunctive procedures; CEUS, contrast-enhanced ultrasound.

**Table 5:** Case series: post-procedural events, complications, recurrences and reinterventions.

Author	Complication < 30 Days Overall	Minor Complications % (N)	Major Complications % (N)	Procedure Related Complications % (N)	Comments	Deaths < 30 Days % (N)	Recurrences % (N)	N Reinterventions	N Expectant or Palliative	Reinterventions Success % (N)	Recurrences-Comments
Golzarian [11]	8.60%	14.3% (1)	14.3% (1)	NA	leg paresis, hemodialysis (recovered) sensory deficit (recovered)	0	0	0 *	0 *	-	-
Faries [12]	NA	5.50%	6.80%	NA	complications overall (not only embolization related)	0	0	0	0	-	-
Maldonado [13]	NA	NA	4.2% (1)	NA	colon ischemia and sepsis (death)	4.2% (1)	25% (6)	3	3 expectant	66.7% (2/3)	1 failed reintervention, underwent open conversion
Choi [14]	NA	NA	NA	NA	multiorgan failure in primary rAAA (death)	14% (1)	42.8% (3)	NA	NA	-	-
Henrikson [15]	NA	NA	16.6% (1)	0%	renal chimney and leg extensions occlusion, leg ischemia + renal failure (death)	0	NA	NA	NA	-	
Chun [16]	0%	NA	NA	NA	-	0	0	0	0	-	
Eberhardt [17]	0%	NA	NA	NA	-	0	12.5% (1)	1	NA	100% (1/1)	1 failed re-embololization, endoanchors, success
Ameli-Renani [18]	24% (6)	4.0% (1)	8% (2)	12% (3)	puncture site hematomas (conservative or surgical revision) LEA dislocation (intervention, recovered)	0	28% (7)	5	2 palliative	60% (3/5)	5 reinterventions including 2 EVAS
Graif [19]	NA	NA	NA	NA	-	0	0	0	0	-	for ELIA no recurrences
Marcelin [20]	0%	NA	NA	NA	-	0	11.1% (1)	1	0	100% (1/1)	
Ierardi [21]	0%	NA	NA	NA	-	0	0	0	0	-	
Marchiori [22]	13.5% (3)	0%	4.5% (1)	9% (2)	LEA disclocation (intervention, recovered) chymney occlusion (intervention, recovered) acute coronary syndrome (death)	4.5% (1)	38% (8)	6	4 (3 palliative, 1 refused)	50% (3/6)	1 failed reintervention, underwent FEVAR, success

Legend: N, number of patients; NA, not available; rAAA, ruptured abdominal aortic aneurysm; FEVAR, fenestrated endovascular aneurysm repair; EVAS, endovascular aneurysm sealing; LEA, liquid embolic agent; ELIA, Type Ia endoleak; *, data specifically referring to ELIA patients.

**Table 6:** Case series: outcomes parameters, follow up methods and timing, follow-up events.

Author	Outcome	Follow-Up Method	Follow-Up Length (Mean) [Months]	Follow-Up Protocol	Freedom from Sac Enlargement % (N)	Comment Sac Enlargement	Freedom from ELIA % (N)	Conversions	N Ruptures in Follow-Up (Time)	Comment Ruptures	Follow-Up Survival N (Non- Aneurysm Related Deaths)
Golzarian [11]	Sac	CT	4–9 (7)	CT within 1 week and every 2 months	100%		100%	0	0		NA
Faries [12]	Sac	CTA	1–60 (24.5)	CTA at 1–6–12, yearly	100%		NA	0	0		NA
Maldonado [13]	Angio	CT	0–40 (nBCA * mean 5.9, coils * means 25)	CTA within 1–6–12, yearly	100% *		92.30%	1	1 (6 months)	refused reintervention (death)	(2)
Choi [14]	Sac EL	CTA	0–53 (18)	CTA at 3–6–12, yearly	83.3% (5/6)	treated with open conversion (n = 1)	NA	0	0		(2)
Henrikson [15]	NA	CT	3–18	CT before discharge and after 1 month	NA		NA	1	1 (18 months)	stent-graft migration and ELIA recurrence, open conversion, recovery	(1)
Chun [16]	Sac EL	CT Duplex	1–10	vary	100%	50% of patients follow-up with duplex	NA	0	0		0
Eberhardt [17]	Angio Sac EL	CT Duplex	8–14	CTA at 6–12 months duplex at 3–6–12, yearly	100%		100%	0	NA		NA
Ameli-Renani [18]	Angio	CT Duplex	0–44.6 (10.2)	NA	85%		80%	0	3 (4, 5, 15 months)	ELIA recurrence, not suitable for further interventions	(1)
Graif [19]	Angio EL	CTA Duplex	0–10 *	CTA, duplex if CTA contraindicated	NA		66.7 (4/6)	1	1 (2.5 months)	refused reintervention (death)	NA
Marcelin [20]	EL Sac	CTA	3–35 (16)	CTA at 1–3, 6, 12, yearly	100%	freedom from sac enlargement after reintervention (n = 1)	78% including TEVAR	0	0		(2)
Ierardi [21]	Angio EL Sac	CTA CEUS	12–30 (16.5)	CEUS before discharge CTA 1–6, 12 months, yearly CT or CEUS at 6 months	100%		NA	0	0		(2)
Marchiori [22]	Angio Sac EL	CTA Duplex MRA	0–65 (15.4)	CTA within 1, 6, 12, yearly duplex at 6 months	76%	4 patients failed secondary procedure, 1 refused it	NA	0	1 (6 months)	contained rupture confirmed at CT	(6)

Legend: Sac, freedom from sac enlargement; Angio, absence of endoleak in the completion angiography; EL, freedom from endoleak in follow-up imaging; CT, computed tomography; CTA, computed tomography angiography; duplex, duplex ultrasound imaging; CEUS, contrast-enhanced ultrasound; MRA, Magnetic resonance angiography; nBCA, n-butyl-cyanoacrylate; N, number of patients; NA, not available; TEVAR, thoracic endovascular aneurysm repair; ELIA, Type Ia endoleak; *, data specifically referring to ELIA patients.
